# Multi-Omics Analysis Reveals Clinical Value and Possible Mechanisms of ATAD1 Down-Regulation in Human Prostate Adenocarcinoma

**DOI:** 10.3390/life12111742

**Published:** 2022-10-30

**Authors:** Chun-Chi Chen, Pei-Yi Chu, Hung-Yu Lin

**Affiliations:** 1 Section of Urology, Departments of Surgery, Changhua Christian Hospital, Changhua 500, Taiwan; 2 Department of Post-Baccalaureate Medicine, College of Medicine, National Chung Hsing University, Taichung 402, Taiwan; 3 School of Medicine, College of Medicine, Fu Jen Catholic University, New Taipei City 242, Taiwan; 4 Department of Pathology, Show Chwan Memorial Hospital, Changhua 500, Taiwan; 5 Department of Health Food, Chung Chou University of Science and Technology, Changhua 510, Taiwan; 6 National Institute of Cancer Research, National Health Research Institutes, Tainan 704, Taiwan; 7 Research Assistant Center, Show Chwan Memorial Hospital, Changhua 500, Taiwan

**Keywords:** prostate adenocarcinoma, ATPase Family AAA Domain Containing 1, biomarker, deep deletion, ETS transcription factor ERG, transcription repression, mitochondria, cell cycle, tumor immune microenvironment

## Abstract

Prostate adenocarcinoma (PRAD) is the most common histological subtype of prostate cancer. Post-treatment biochemical recurrence is a challenging issue. ATAD1 (ATPase Family AAA Domain Containing 1) plays a vital role in mitochondrial proteostasis and apoptosis activity, while its clinical value in PRAD and its impact on the tumor microenvironment (TME) remain unanswered. In this study, we aimed to investigate the clinical value and possible mechanisms of ATAD1 in PRAD via multi-omics analysis. Using cBioPortal, we confirmed that ATAD1 alteration was associated with gene expression and unfavorable DFS. Deep deletion predominantly occurred in PRAD. By integrating DriverDBv3 and GEPIA2, we noted ATAD1 downregulation in PRAD tissues compared to normal tissues, associated with unfavorable DFS in PRAD patients. DNA repair genes ATM, PARP1and BRCA2 had positive associations with ATAD1 expression. We found that the generalization value of ATAD1 could be applied to other cancers such as KIRC and UCEC. In addition, LinkedOmics identified that the functional involvement of ATAD1 participates in mitochondrial structure and cell cycle progression. Using TIMER analysis, we demonstrated that ATAD1 downregulation correlated with an immunosuppressive TME. Furthermore, we accessed a GSE55062 dataset on UALCAN and discovered the involvement of ERG-mediated transcriptional repression on ATAD1 downregulation. Cross-association screening of shATAD1 efficacy vs. altered mRNAs identified 190 perturbed mRNAs. Then, functional enrichment analysis using the Metascape omics tool recognized that shATAD1-perturbed mRNAs are primarily in charge of the activation of Wnt/β-catenin pathway and lipid metabolic processes. In conclusion, multi-omics results reveal that ATAD1 downregulation is a clinical biomarker for pathological diagnosis and prognosis for patients with PRAD. Reduced ATAD1 may be involved in the enhanced activity of mitochondria and cell cycle, as well as possibly shaping an immunosuppressive TME. ERG serves as an upstream transcriptional repressor of ATAD1. Downstream mechanisms of ATAD1 are involved in Wnt/β-catenin pathway and lipid metabolic processes.

## 1. Introduction

Prostate adenocarcinoma (PRAD) is the most common histological subtype of prostate cancer [1]. Prostate cancer occupies the second most common malignant tumor and the fifth major cause of cancer mortality in men globally [2]. With advancements in detection and treatment, patients with PRAD at an early stage had an 99% ten-year overall survival (OS) rate [3]. Active surveillance, focal therapy, radiotherapy, radical prostatectomy, androgen restriction, as well as more recently, immunotherapy, are all available as treatments for metastatic illness [4,5]. However, after having treatment without signs of overt metastatic illness, more than half of patients will develop biochemical recurrence (BCR) [6], which is characterized by elevated prostate-specific antigen (PSA) levels. In regard to anti-tumor immunity, prostate cancer is often defined as a “cold tumor” with an immunosuppressive tumor microenvironment (TME), in which tumor-infiltrating lymphocytes (TILs) may exacerbate the development of prostate cancer [7]. TILs from prostate cancer biopsy samples are predisposed to the Tregs and T helper 17 (Th17) phenotypes, which block autoreactive T cells and antitumor immune responses [8]. Thus, the identification of biomarkers that features good clinicopathological value is an urgent need for the prognosis and companion diagnosis of these patients.

ATAD1 (ATPase Family AAA Domain Containing 1) plays a vital role in many biological scenarios, including preserving mitochondrial proteostasis [9,10] and activating apoptotic pathways via depleting BCL-family protein [11]. In addition, ATAD1 was recently found to participate in neurological development, such as the regulation of synaptic plasticity. For now, the best understood function of ATAD1 is the removal of mistargeted tail-anchored (TA) proteins from the mitochondrial outer membrane [12]. Interestingly, the clinical value of ATAD1 in PRAD and its impact on TME remain unanswered.

In this study, we aimed to investigate the clinical value of ATAD1 in PRAD via multi-omics analysis. Firstly, the mutational profile of ATAD1 and clinical significance of its genetic alteration were examined. Secondly, its predominant implication in the diagnosis and prognosis of PRAD were identified. Thirdly, the possible mechanisms behind ATAD1 downregulation on PRAD progression were determined. Fourthly, the association between ATAD1 expression and the prostate microenvironment at the single-cell level and its relationship with immune infiltration were analyzed. Finally, we found that ERG may act as a transcriptional repressor on the gene expression of ATAD1. 

## 2. Materials and Methods

### 2.1. cBioPortal

cBioPortal is gear toward interactive analysis of genomics and clinical profiles in human tumor tissue from The Cancer Genome Atlas (TCGA) databases [13]. The OncoPrint module was used to examine the profiled for mutations, mutation spectrum, mutation counts, tumor mutational burden (TMB), and genetic alterations of ATAD1 across cancers. The Mutations modules was used to analyze a lollipop plot of gene mutation landscape. Log2 copy-number values from Affymetrix SNP6 was selected to analyze the correlation coefficient of copy number with mRNA levels. The Comparison/Survival module was used to analyze survival rate based on gene alteration status. The Cancer type panel of the Cancer Types Summary module was employed to genetic alteration frequency across all cancers. 

### 2.2. DriverDB

DriverDBv3 is a cancer driver gene database that provides integrative multi-omics analysis bioinformatics algorithms to identify driver genes/mutations [14]. The Visualization panel of the CNV module was used to determine the relations between the top-ranked genes and their CNV in PRAD samples. The Locus enrichment panel was used to illustrate the loci of genes within all chromosomes and the correlation coefficient value of CNV and mRNA levels. The Visualization panel of the Expression module was utilized to exhibit ATAD1 gene expression levels in normal and PRAD specimens. 

### 2.3. GEPIA2

The updated version of the Gene Expression Profiling Interactive Analysis (GEPIA2) public database was designed to deliver fast and customizable functionalities based on TCGA and GTEx datasets, and features 198,619 isoforms and 84 cancer subtypes [15]. The Survival Analysis module was used to determine the probability of disease-free survival (DFS) based on the expression levels of a gene. The cutoff value of the gene expression level was set at 25–75% (high–low).

### 2.4. Human Protein Atlas

The differential expression levels of ATAD1 protein in normal and tumor tissues was validated using the Human Protein Atlas portal [16,17,18], which is geared toward mapping the biology of all human proteins in cells, tissues, and organs by integrating a variety of biological techniques, including antibody-based imaging, mass spectrometry-based proteomics, single-cell level transcriptomics, and systems.

### 2.5. Tumor Immune Estimation Resource (TIMER)

TIMER is a public web server for comprehensive evaluations of the clinical impact of different immune cells in diverse cancer types [19]. Its updated version TIMER2.0 exploits multiple algorithms to estimate infiltration levels of immune cells [20]. We used the Gene_DE and the Gene_Corr module to analyze ATAD1 expression in pan-cancer view and investigate the associations between immune infiltrates and genetic markers, respectively.

### 2.6. LinkedOmics

LinkedOmics is a multi-omics web portal comprising 32 cancer types and a total of 11,158 patients from TCGA project [21]. The LinkFinder module was used to analyze and visualize the associations between ATAD1 and other attributes for the PRAD cohort. The LinkInterpreter module was used to perform Gene Set Enrichment Analysis (GSEA), whereby identified associations can be transformed into biological understanding through gene ontology (GO) and pathway. The Rank Criteria exploited *p*-value. The Minimum Number of Genes Size and Simulation were set at 3 and 500, respectively.

### 2.7. Chromatin Immunoprecipitation Sequencing (ChIP-Seq) Dataset

The ChIP-Seq dataset derived from GSE55062, GSE83653 and GSE73616 in which ERG ChIP-seq was performed in human prostate cancer cell line VCaP [22]. The binding of ERG to ATAD1 promote region was analyzed on The University of Alabama at Birmingham CANcer (UALCAN) data analysis portal, which allows users to perform in silico validation of potential genes of interest [23,24].

### 2.8. Statistical Analysis

Statistical methods were as previously described [25]. Briefly, quantitative data were analyzed using unpaired t-test for two-group comparison or one-way analysis of variance (ANOVA) for three-or-more-group comparison, as appropriate. The Pearson method was employed to analyze correlations. The Kaplan–Meier (log rank) test was used to give the significance of P value for the difference between two groups. 

## 3. Results

### 3.1. ATAD1 Alteration Is Associated with Gene Expression and Unfavorable Clinical Outcome

**Figure 1:**
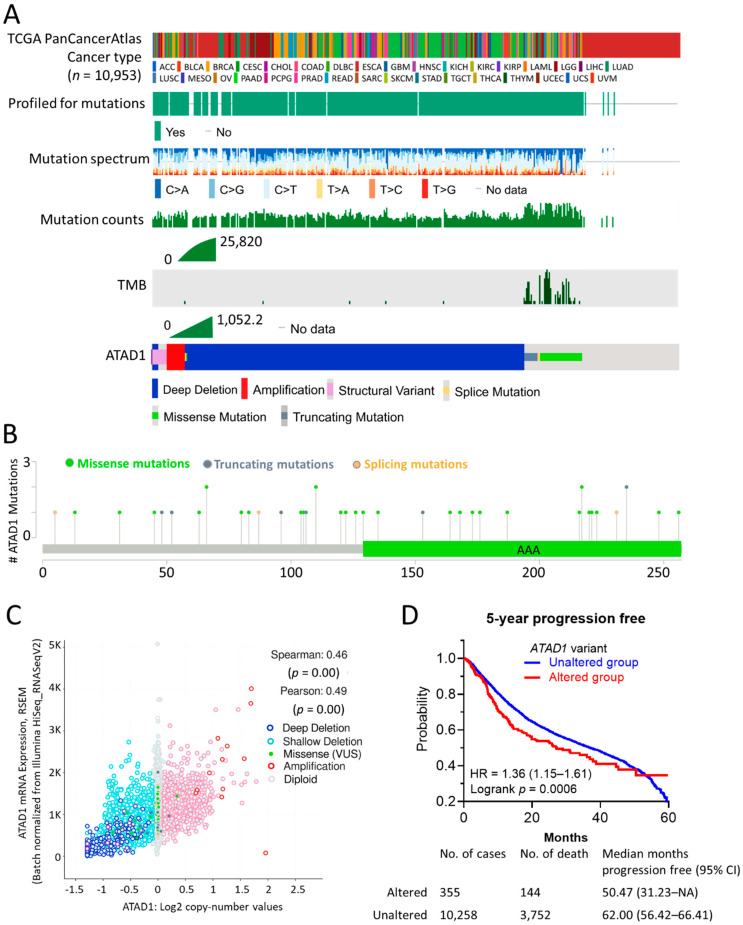
Mutation Profile and clinical relevance of ATAD1: (**A**) the profiled for mutations, mutation spectrum, mutation counts, tumor mutational burden (TMB), and genetic alterations of ATAD1 across patients (*n* = 10,953) of all TCGA PanCancerAtlas cancer types; (**B**) lollipop plot illustrating number and the distribution of the mutations spanning ATAD1 amino acid sequence. AAA, ATPase family associated with various cellular activities; (**C**) correlation analysis of ATAD1 mRNA expression levels with ATAD1 copy number in 9889 samples from 32 studies; and (**D**) Kaplan–Meier analysis of the 5-year disease-free survival rate based on altered/unaltered ATAD1 variant. HR, hazard ratio. AML, acute myeloid leukemia; ACC, adrenocortical carcinoma; BLCA, bladder urothelial carcinoma; LGG, brain lower grade glioma; BRCA, breast invasive carcinoma; CESC, cervical squamous cell carcinoma and endocervical adenocarcinoma; CHOL, cholangiocarcinoma; CML, chronic myelogenous leukemia; COAD, colon adenocarcinoma; ESCA, esophageal carcinoma; GBM, glioblastoma multiforme; HNSC, head and neck squamous cell carcinoma; KICH, kidney chromophobe; KIRC, kidney renal clear cell carcinoma; KIRP, kidney renal papillary cell carcinoma; LIHC, liver hepatocellular carcinoma; LUAD, lung adenocarcinoma; LUSC, lung squamous cell carcinoma; DLBC, lymphoid neoplasm diffuse large b-cell lymphoma; MESO, mesothelioma; OV ovarian serous cystadenocarcinoma; PAAD, pancreatic adenocarcinoma; PCPG, pheochromocytoma and paraganglioma; PRAD, prostate adenocarcinoma; READ, rectum adenocarcinoma; SARC, sarcoma; SKCM, skin cutaneous melanoma; STAD, stomach adenocarcinoma; TGCT, testicular germ cell tumors; THYM, thymoma; THCA, thyroid carcinoma; UCS, uterine carcinosarcoma; UCEC, uterine corpus endometrial carcinoma; UVM, uveal melanoma.

The mutational landscape of ATAD1 in cancer samples of 10953 patients was analyzed on cBioPortal web server (Figure 1A). The profiled for mutation, mutation spectrum and mutation counts went hand-in-hand with the tumor mutational burden (TMB). ATAD1 genetic alterations comprised deep deletion, amplification, structural variant, splice mutation, missense mutation, and truncating mutation. Deep deletion occupies most of the genetic alteration types of ATAD1. Notably, the occurrence of missense, truncating and splice mutations was in synchronicity with TMB. The number and the distribution of the mutations across the amino acid sequence of ATAD1 is illustrated in Figure 1B. The correlation coefficient analysis revealed that the gene expression level of ATAD1 has a positive association with the copy number (Figure 1C). To confirm whether ATAD1 alteration is associated with clinical outcome, we employ Kaplan–Meier analysis to examine the 5-year progression free survival rate of patients harboring altered (*n* = 144) or unaltered ATAD1 (*n* = 10,258) in the tumor tissue. As shown in Figure 1D, ATAD1 altered group had a shorter 5-year progression free survival compared to ATAD1 unaltered group (HR = 1.36, *p* = 0.0005). These findings indicate the involvement of ATAD1 alteration in the gene expression and unfavorable clinical outcome of cancer patients.

### 3.2. Deep Deletion of ATAD1 Predominantly Occurs in PRAD

**Figure 2:**
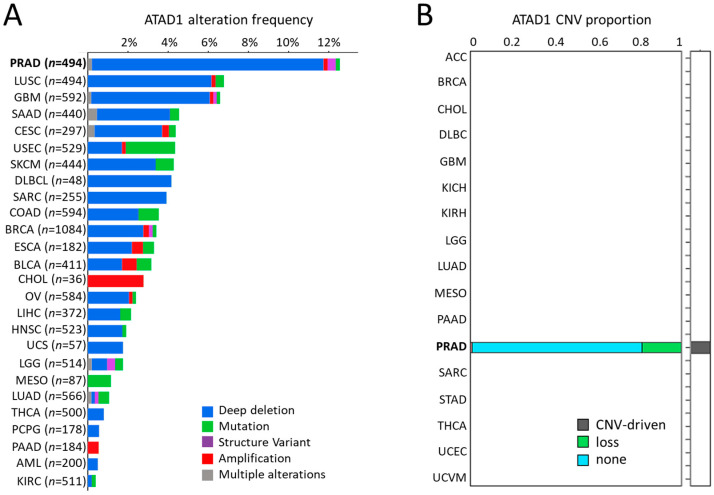
Deep deletion of ATAD1 predominantly occurs in PRAD among cancers: (**A**) the alteration frequency of ATAD1 across all cancers; and (**B**) bar chart illustrating the copy number variation (CNV) proportion of ATAD1 in various cancer types, which is analyzed by two CNV tools: iGC and diggit. The sample proportion is shown in the plot when the cancer is identified by one or both of the tools. Note that ATAD1 was identified as a CNV-driven gene particularly in PRAD.

Next, we examined the significance of ATAD1 across different cancers. PRAD showed the highest ATAD1 alteration frequency compared to other cancer types, in which deep deletion occupied the majority (Figure 2A). Similarly, ATAD1 copy number variation (CNV) predominantly occurs in PRAD is well shown by employing iGC [26] and digit [27] R packages (Figure 2B).

### 3.3. Diagnostic and Prognostic Value of ATAD1 Down-Regulation in PRAD

**Figure 3:**
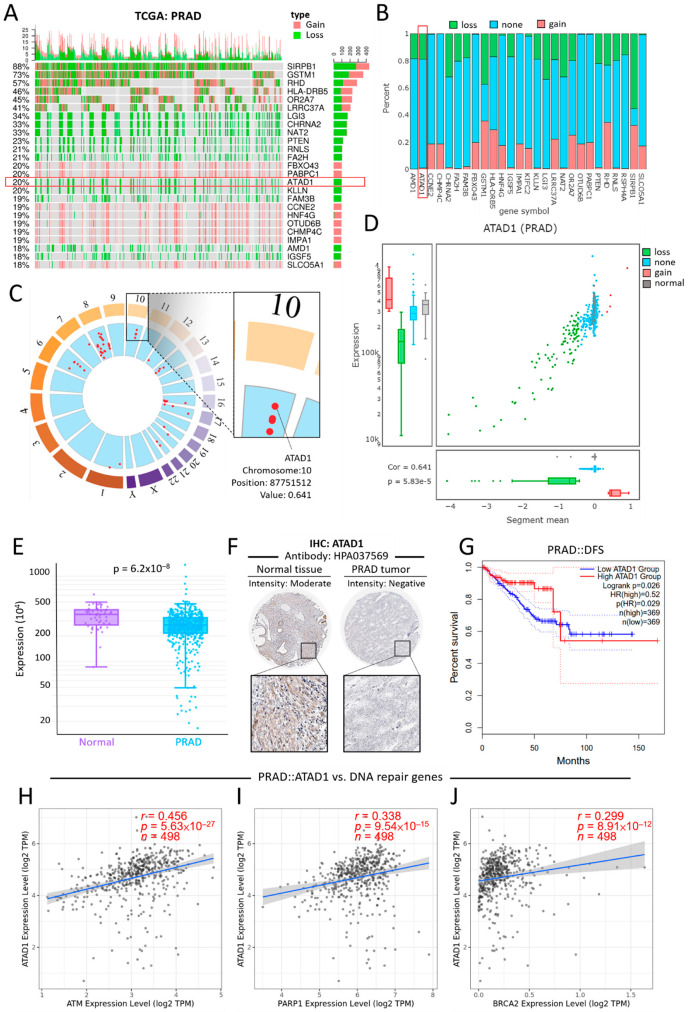
Deletion-associated downregulation of ATAD1 features clinical value on the differential gene expression, prognostic prediction and pathological testing (**A**) oncoplot illustrates the relations between the top 25 genes and their CNV in patients with PRAD; the samples of cancer patients are on the x-axis and the top 25 genes are on the y-axis. Note that ATAD1 contains only CNV loss and non-alteration; (**B**) bar chart provides an overview of the CNV percentages for each of the top 25 genes in patients with PRAD; (**C**) locus enrichment graph shows the loci of all listed genes within all chromosomes. Each red dot represents a gene and its related position on the chromosome. Enlarged square shows the chromosome, position, and value of ATAD1. The value represents the correlation between mRNA expression and CNV; (**D**) a combination of scatter plot and boxplots shows a detailed view of the CNV distribution and correlation of ATAD1 in PRAD; (**E**) box plot shows the ATAD1 expression levels in normal tissues and PRAD tumors. (**F**), Immunohistochemistry staining using antibody HPA037569 for the detection of ATAD1 protein expression levels in normal tissues and PRAD tumors; (**G**) Kaplan–Meier analysis of the disease-free survival rate based on low/high ATAD1 expression. HR, hazard ratio; and (**H**–**J**) Spearman correlation analysis of ATAD1 with DNA repair genes for prostate cancer such as ATM (**H**) and PARP1 (**I**) and BRCA2 (**J**).

The relationship between the top 25 altered genes and their CNV in patients with PRAD was shown using an oncoplot (Figure 3A). There were only six genes that have association with clinical outcome, including ATAD1, CHRAN2, FA2H, FBXO43, LGI3 (Supplementary Figure S1). Specifically, ATAD1 contains mostly CNV loss and non-alteration (Figure 3B). Locus enrichment located ATAD1 position on chromosome 10 and revealed positive correlation with mRNA expression levels (Figure 3C). In PRAD samples, ATAD1 CNV correlated positively with mRNA expression levels (Figure 3D). Compared to normal tissues, PRAD tissues showed a decreased expression level of ATAD1 mRNA (Figure 3E) and ATAD1 protein (Figure 3F). Low gene expression of ATAD1 was associated with decreased disease-free survival rate (DFS) (Figure 3G). As the genetic aberrations can be due to the alteration of several key genes that are responsible for DNA repair, including ATM, PARP1/2, BRCA1/2 [28], we further looked into the impact of DNA repair genes on ATAD1 expression. As shown in Figure 3H–J, ATAD1correlated positively with ATM, PARP1and BRCA2. Together, these findings indicate that the downregulation of ATAD1 can act as a marker for the diagnosis and prognosis of PRAD and its genetic alteration is associated with perturbation of intracellular DNA repair. The alteration frequency and CNV proportions used are robust to claim the results.

### 3.4. Generalization Value of ATAD1 

**Figure 4:**
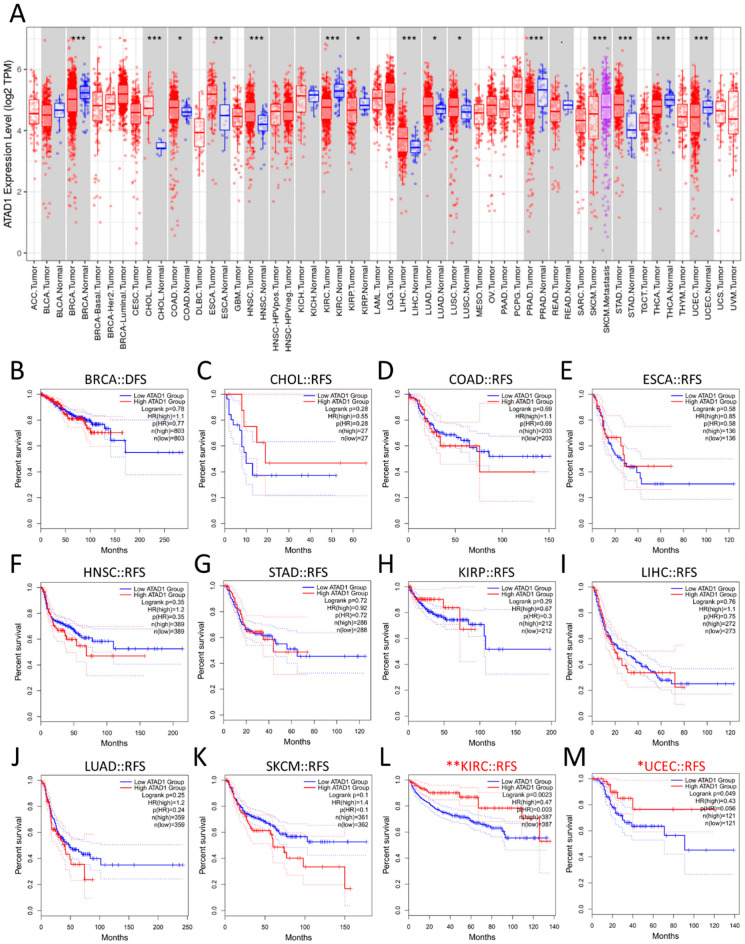
Generalization value of ATAD1 across cancers (**A**); expression profile of ATAD1. * *p* < 0.05, ** *p* < 0.01, *** *p* < 0.001 between a particular tumor and corresponding normal tissue (**B**–**M**); Kaplan–Meier survival analysis representing the probability of disease-free survival (DFS) based on low/high ATAD1 expression in breast cancer (BRCA) (**B**); cholangiocarcinoma (CHOL) (**C**); colon adenocarcinoma (COAD) (**D**); esophageal carcinoma (ESCA) (**E**); head and neck squamous cell carcinoma (HNSC) (**F**); stomach adenocarcinoma (STAD) (**G**); kidney renal papillary cell carcinoma (KIRP) (**H**); liver hepatocellular carcinoma (LIHC) (**I**); lung adenocarcinoma (LUAD) (**J**); skin cutaneous melanoma (SKCM) (**K**); kidney renal clear cell carcinoma (KIRC) (**L**); and uterine corpus endometrial carcinoma (UCEC) (**M**). * *p* < 0.05 and ** *p* < 0.01 calculated by log-rank test comparing low ATAD1 and high ATAD1 group.

To verify whether ATAD1 has a broad value, we investigated differential expression and examined DFS across cancers. We observed that a number of cancers, apart from PRAD, show altered ATAD expression, such as breast invasive carcinoma (BRCA), cholangiocarcinoma (CHOL), colon adenocarcinoma (COAD), esophageal carcinoma (ESCA), head and neck squamous cell carcinoma (HNSC), kidney renal clear cell carcinoma (KIRC), kidney renal papillary cell carcinoma (KIRP), liver hepatocellular carcinoma (LIHC), lung adenocarcinoma (LUAD), lung squamous cell carcinoma (LUSC), skin cutaneous melanoma (SKCM), stomach adenocarcinoma (STAD), thyroid carcinoma (THCA), and uterine corpus endometrial carcinoma (UCEC) (Figure 4A). On the other hand, the DFS of most cancers had no association with ATAD1 expression (Figure 4B–K), while low ATAD1 was associated with poor DFS in KIRC (Figure 4L) and UCEC (Figure 4M). Therefore, ATAD1 downregulation could feature clinical value on KIRC and UCEC.

### 3.5. Functional Involvement of ATAD1 in Mitochondrial Structure and Cell Cycle Progression

**Figure 5:**
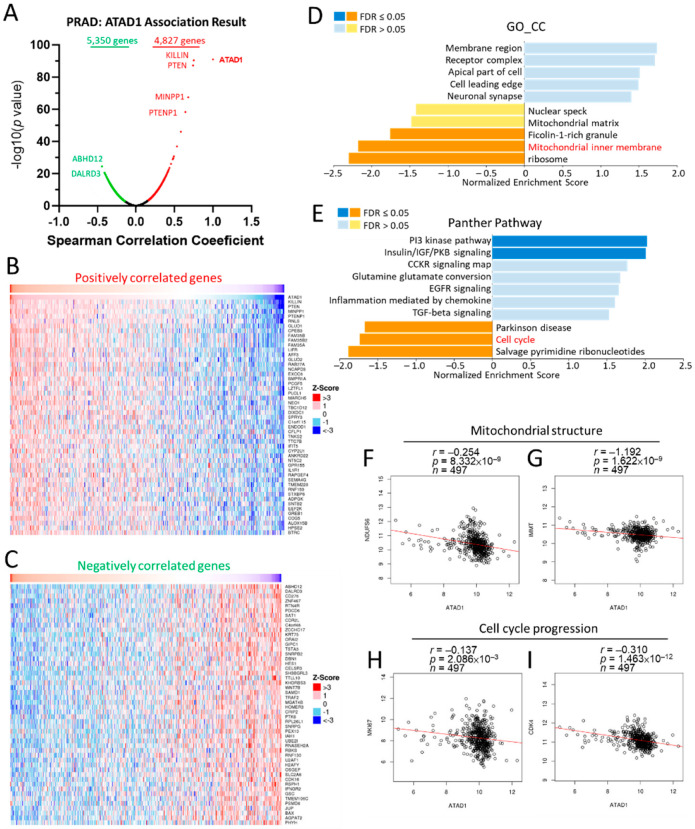
Involvement of ATAD1 in mitochondrial structure and cell cycle progression in PRAD. Enrichment analysis of TCGA-PRAD samples was conducted by using the LinkedOmics functional modules. (**A**), Volcano plot showing the Pearson correlation coefficient and p value of the ATAD1-coexpressed genes; (**B**,**C**) heatmap showing top 50 positively (**B**) and negatively (**C**) correlated genes with ATAD1; (**D**,**E**) bar chart showing the normalized enrichment score, involved gene count and false discovery rate (FDR) of the gene ontology cellular component (GO_CC) terms (**D**) and Panther pathway terms (**E**); (**F**,**G**) dot pots showing the correlation between ATAD1 and mitochondrial structure genes such as NDUFS6 (**F**) and IMMT (**G**); (**H**,**I**) dot pots of the correlation between ATAD1 and cell cycle progression genes such as MKI67 (**H**) and CDK4 (**I**).

To gain more insight into the biological roles of ATAD1, we subsequently employed the functional modules in LinkedOmics [21], which permits the examination of co-expressed genes and analysis of functional enrichment for the TCGA-PRAD cohort. A total of 4827 genes showed significantly positive correlations with ATAD1, while 5350 genes had significant negative correlations with ATAD1 (Figure 5A). Heap maps demonstrated top 25 genes with the most significant positive (Figure 5B) and negative correlations (Figure 5C) with ATAD1. We noted that the significant enrichment of inhibited terms comprised ficolin-1-rich granule, mitochondrial inner membrane and ribosome in gene ontology cellular component (GO_CC) (Figure 5D) and Parkinson disease, cell cycle and salvage pyrimidine ribonucleotides in Panther pathway (Figure 5E). Considering that ATAD1 localized to the nucleoli rim and mitochondria (Supplementary Figure S2), we accordingly analyzed the relationship between ATAD1 and mitochondrial structure and cell cycle progression. Pearson’s correlation analysis showed negative correlations of ATAD1 with mitochondrial genes such as NDUFS6 (Figure 5F) and IMMT (Figure 5G) and cell cycle genes such as MKI67 (Figure 5H) and CDK4 (Figure 5I).

### 3.6. Correlation of ATAD1 Down-Regulation with Immunosuppressive TME

**Figure 6:**
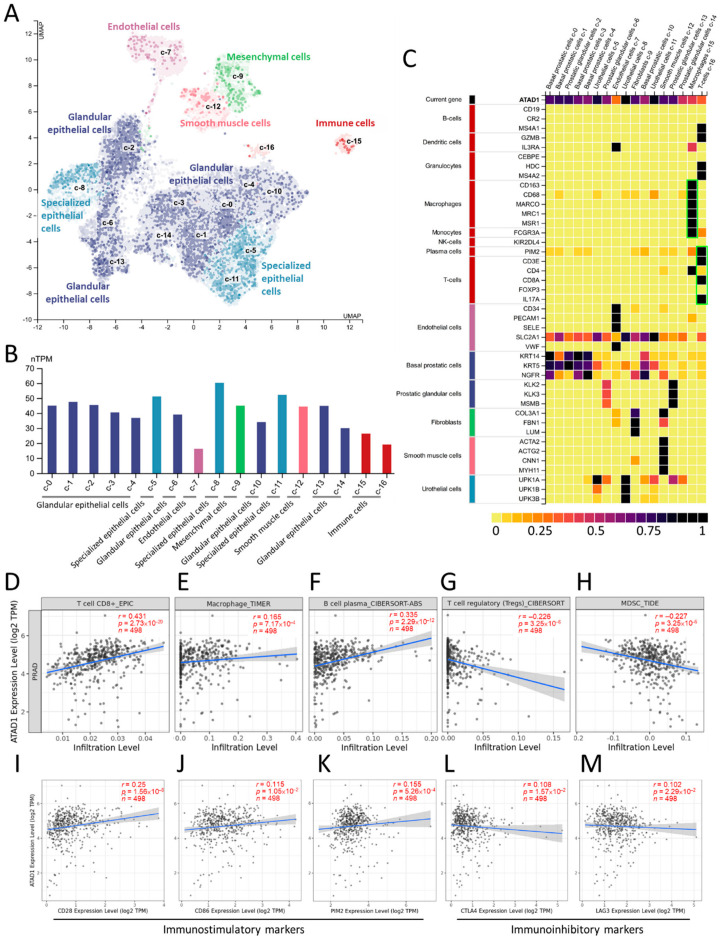
The correlation between ATAD1 and immune microenvironment: (**A**,**B**) UMAP plots (**A**) and bar chart (**B**) showing the single cell-RNA sequencing that identifies the single cell type clusters in prostate tissue; (**C**) heatmap showing the expression of ATAD1 gene and iconic markers in different single cell types clusters in prostate tissues. Note that ATAD1 presents relatively low levels compared to markers of macrophage, plasma cells and T cells; (**D**–**H**) dot plots showing the correlation between ATAD1 and infiltrated immune cells including CD8^+^ T cells (**D**), macrophages (**E**), B cells (**F**), Tregs (T cells regulatory) (**G**), and MDSCs (myeloid derived suppressor cells) (**H**); (**I**–**M**) dot plots showing the correlation between ATAD1 and immunostimulatory markers such as CD28 (**I**), CD86 (**J**) and PIM2 (**K**), and immunoinhibitory markers such as CTLA4 (**L**) and LAG3 (**M**).

Considering the vital role of TME in tumor growth, spread, and escape from immune-mediated destruction [29,30], we further analyzed the relationship between ATAD1 and tumor-immune interaction. We used single-cell RNA sequencing datasets to analyze the transcriptomic profile and visualize the heterogenous cell populations. The 10× Genomics datasets retrieved from HPA portal was used to explore the potential association of ATAD1 with immune cells in the prostate microenvironment. We acquired gene expression data of 35,862 single cells to construct UMAP plot and bar chart in which the specificity and distribution of ATAD1 in different cell populations in the prostate were analyzed to determine the differential expression at single-cell level (Figure 6A,B). The heatmap demonstrated the expression of ATAD1 and iconic biomarkers of multiple cell clusters in prostate tissue (Figure 6C), whereby we identified specific immune cells (green boxes) such as macrophage, plasma cells and T cells that harbor high expression from the single-cell sequencing data. Accordingly, we further analyzed the correlation of ATAD1 with the infiltrated immune cells. ATAD1 positively correlated with the infiltration level of CD8+ T cell (Figure 6D), macrophage (Figure 6E), and plasma cells (Figure 6F), while negatively correlated with that of regulatory T cells (Tregs) (Figure 6G) and myeloid derived suppressor cells (MDSC) (Figure 6H). Furthermore, we noted that ATAD1 had positive associations with immunostimulatory markers such as CD28 (Figure 6I), CD86 (Figure 6J) and PIM2 (Figure 6K), while showed negative correlations with immunosuppressive markers such as CTLA4 and (Figure 6L) LAG3 (Figure 6M). Taken together, these results suggest that ATAD1 downregulation is associated with an immunosuppressive TIME. 

### 3.7. ERG-Mediated Transcriptional Repression Is Involved in ATAD1 Downregulation

**Figure 7:**
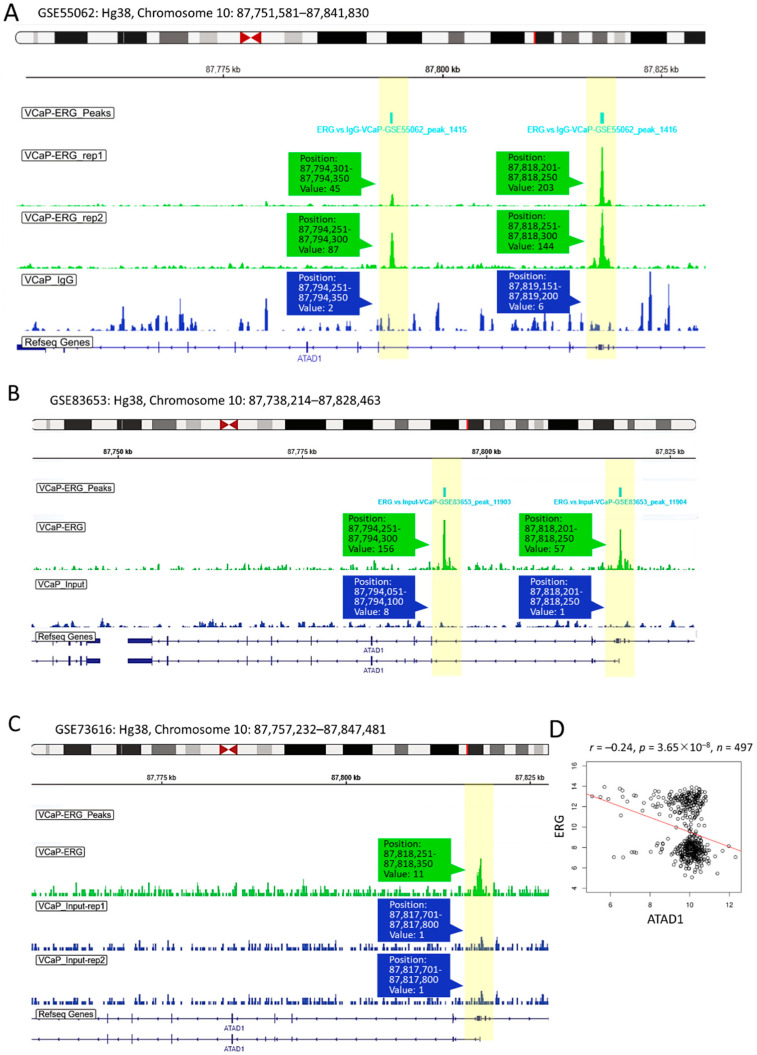
Upstream transcriptional repression of ATAD1 by ERG: ChIP-Seq (chromatin immunoprecipitation sequencing) showing interactivity between ATAD1 promoter and ERG (**A**–**C**), The ERG ChIP-Seq data retrieved from GSE55062 (**A**); GSE83653 (**B**); and GSE73616 (**C**) analyzing the association of ERG with ATAD1 promoter on chromosome 10 in prostate cancer cell line VCaP. Immunoprecipitated lysates treated with anti-ERG or IgG (as negative control) undergo sequence analysis on Illumina HiSeq 1000 platform. The yellow bars highlight the peak signal of ChIP-seq in which the position and readout value are shown; (**D**) dot plot showing the Pearson correlation coefficient of ATAD1 with ERG expression levels retrieved from TCGA-PRAD dataset.

As ERG acts as an oncogenic transcription factor in PRAD by repressing tumor suppressor such as PTEN [31], we herein asked whether ERG exerts transcriptional repression on ATAD1 in PRAD. To address this, we examined the interactivity between the ATAD1 promoter and ERG by accessing the GSE55062 dataset that conducted chromatin immunoprecipitation (ChIP) assay in human prostate cancer cell line VCaP [22]. As shown in Figure 7A, anti-ERG ChIP-seq showed positive signal on two ATAD1 promoter regions compared to isotype IgG, indicating the interactivity between ERG and ATAD1 promoter. Similar results observed in GSE83653 (Figure 7B) and GSE73616 (Figure 7C) were robust to claim the ERG-ATAD1 interactivity result. Moreover, we noted that ERG negatively correlated with ATAD1 expression (Figure 7D), supporting the notion that ERG exerts transcriptional repression on the ATAD1 expression. 

**Figure 8:**
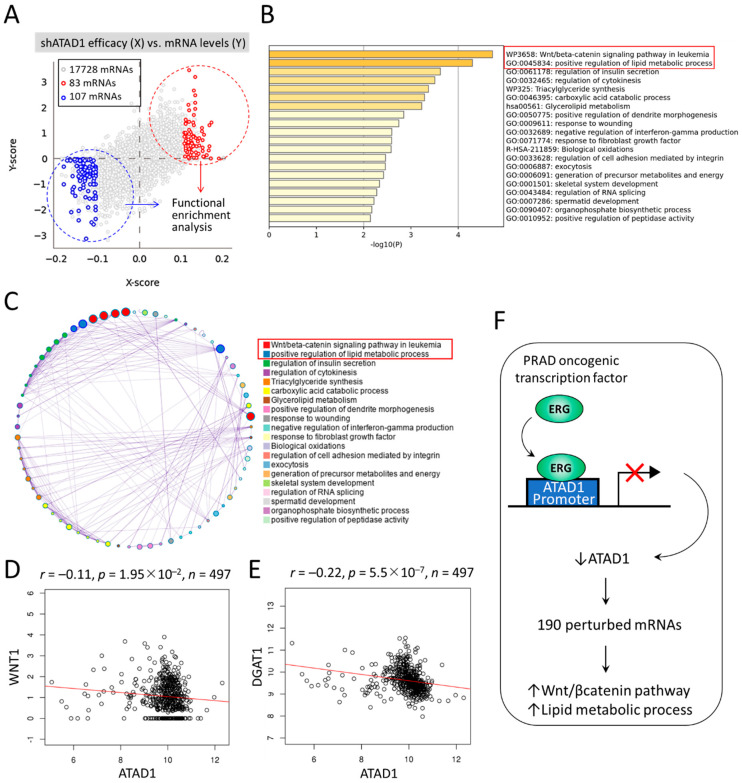
Downstream mechanisms of ATAD1 involved in Wnt/beta-catenin signaling and lipid metabolic process: (**A**) dot plots showing the cross-association screening between shATAD1 efficacy and altered mRNA levels. X-score represents log(fold-change) of shATAD1 efficacy between samples of high and low expression of target mRNA. Y-score represents log(fold-change) of target mRNA levels between samples of high and low shATAD1 efficacy. Red and blue dots denote significantly upregulated and downregulated mRNAs by shATAD1, respectively. Then the 190 altered mRNAs are subjected to functional enrichment analysis using Metascape portal; (**B**) bar chart of enriched terms across input mRNAs, colored by p-values. Top two enriched terms (*p* < 10^−4^) are highlighted; (**C**) Network of enriched terms colored by cluster ID, where nodes that share the same cluster ID are typically close to each other; (**D**,**E**), dot plot showing the Pearson correlation coefficient of ATAD1 with WNT1 (**D**) and DGAT1 (**E**) by retrieving TCGA-PRAD datasets; and (**F**) a schematic illustrates that oncogenic ERG acts as a transcriptional repressor to reduce ATAD1 expression, which could lead to 190 altered mRNAs and perturbation of Wnt/beta-catenin pathway and lipid metabolic process.

To gain insight into the downstream mechanisms of ATAD1, we sought to dissect the perturbed functional pathways following ATAD1 knockdown in urinary cancer cell lines. We firstly examined the cross-association between shATAD1 efficacy and altered mRNA levels using Q-omics software program [32]. Amid the 17,728 mRNAs, 83 and 107 mRNAs were identified to be respectively upregulated and downregulated by shATAD1 (Figure 8A). Then, the perturbed 190 mRNAs were subjected to functional enrichment analysis using Metascape portal [33]. The top two enriched terms were noted: Wnt/beta-catenin signaling pathway and positive regulation of lipid metabolic process (Figure 8B). The relationship between the enriched terms was illustrated by a network plot (Figure 8C). The negative correlations of ATAD1 with WNT1 (Wnt family member 1) and with the key gene of lipid metabolism DGAT1 (diacylglycerol O-acyltransferase 1) were well shown by accessing TCGA-PRAD data repository (Figure 8D,E), suggesting a negative role of ATAD1 in the activity of Wnt signaling and lipid metabolism. In light of these results, we proposed that ERG acts as a transcriptional repressor to reduce ATAD1 expression, which could lead to the activation of Wnt/β-catenin pathway and lipid metabolic process (Figure 8F).

## 4. Discussion

**Figure 9:**
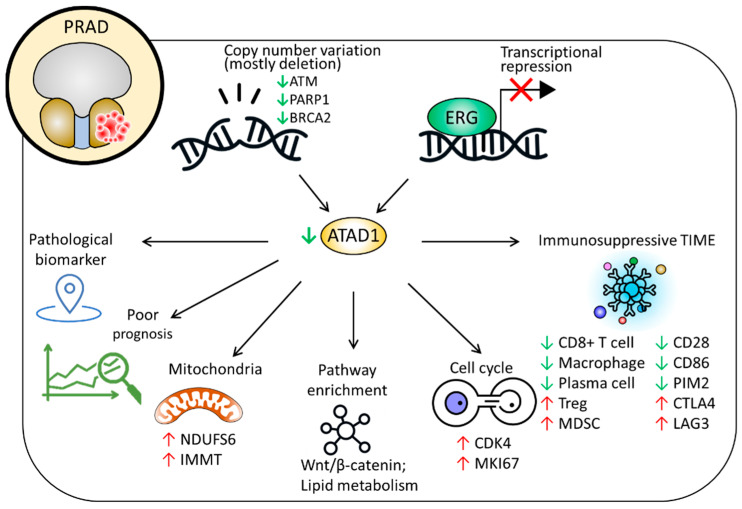
Proposed model delineating the clinicopathological implications and possible roles of ATAD1 in PRAD.

Although the biological function of ATAD1 in mitochondrial proteostasis is emerging, clinicopathological value of ATAD1 in patients with PRAD remains unclear. In this study, we unveiled that decreased ATM, PARP1 and BRCA2 are associated with ATAD1 downregulation in PRAD tissues. In addition, the transcription repression of ERG may also play a role. ATAD1 downregulation could serve as a biomarker for pathology and poor prognosis. The downstream of ATAD1 is involved in the regulation of Wnt/β-catenin pathway and lipid metabolic process. ATAD1 has a negative association with functional markers of mitochondria (NDUFS6 and IMMT) and cell cycle (CDK4 and MKI67). Moreover, reduced ATAD1 correlates with an immunosuppressive TME. A proposed model is summarized in Figure 9. 

To the best of our knowledge, report in regard to the clinical value of ATAD1 remains scarce. A preprint study reported that patients with ATAD1-null prostate tumor had favorable overall survival and that loss of ATAD1 in PC3 cells sensitized to the treatment of Bortezomib, while overexpression of ATAD1 developed drug resistance [11]. In contrast, our study demonstrates that PRAD tissues show ATAD1 downregulation compared to normal tissue and that low ATAD1 indicates unfavorable DFS. However, a major caveat of this study lies in the lack of an experimental study that exploits genetic manipulation to verify the biological role of ATAD1 in the cancerous behaviors of PRAD. Therefore, further study is warranted to clarify the impact of ATAD1 alteration.

While certain metastatic malignancies, like melanoma, lung cancer, and renal cell carcinoma, have dramatically responded to immunotherapy, prostate cancer has typically failed to demonstrate a meaningful response [7]. In fact, with an immunosuppressive microenvironment, prostate cancer is frequently described as a “cool” tumor. Sfanos et al. have shown that TILs from prostate cancer biopsy samples are predisposed to the Tregs and T helper 17 (Th17) phenotypes, which block autoreactive T cells and antitumor immune responses [8]. In our study, we discovered that ATAD1 downregulation correlates with increased infiltration of Tregs and MDSC and decreased infiltration of CD8+ T cell, macrophage, and plasma cells, which shape an immunosuppressive TME. In addition, we found that reduced ATAD1 is related to decreased immunostimulatory markers and increased immunoinhibitory markers, further underscoring its potential impact in TME. However, reports with regard to whether ATAD1 plays a role behind the mechanisms accounting for shaping of the TME are still lacking. More efforts on this issue are required in the future.

The oncogenic transcription factor ERG was shown to be implicated in cellular homeostasis, survival, differentiation, angiogenesis and vasculogenesis [34]. With regard to prostate cancer, Adamo et al. reported that ERG acts as an oncogenic transcription factor by repressing the activity of tumor suppressor gene such as PTEN [31]. In this study, we identified the direct binding of ERG to ATAD1 promoter and a negative correlation of ERG with ATAD1 in PRAD specimen, indicating that ERG may exert transcriptional repression effect on ATAD1. In addition, the recognition of Wnt/β-catenin signaling and lipid metabolic process provides further insight into the downstream action of ATAD1. Nevertheless, further in vivo study is required to clarify the molecular basis behind this, and test whether ERG, ATAD1, Wnt/β-catenin and lipid metabolism could be therapeutic targets in PRAD.

In conclusion, multi-omics results reveal that ATAD1 downregulation is a clinical biomarker for pathological diagnosis and prognosis for patients with PRAD. Reduced ATAD1 may be involved in the enhanced activity of mitochondria and cell cycle, as well as possibly shaping an immunosuppressive TME.

## Data Availability

The dataset supporting the conclusions of this article is included within the article.

## Supplementary Materials

The following supporting information can be downloaded at: https://www.mdpi.com/article/10.3390/life12111742/s1, Figure S1. Kaplan–Meier survival analysis representing the probability of disease-free survival (DFS) based on high/low gene expression of PRAD patients in TCGA datasets. The cutoff vale is set at 25%–75% (high–low); Figure S2. Representative immunofluorescence image showing intracellular localization of ATAD1 in U-251MG cells. ATAD1 (green) is probed by antibody HPA037569. Nucleus (blue) and microtubule (red) are counterstained. Bottom schematic depicts the ATAD1 localization detected in nucleoli and mitochondria.
