# Supplementary material for: Multi-Omics Analysis Reveals Clinical Value and Possible Mechanisms of ATAD1 Down-Regulation in Human Prostate Adenocarcinoma
Source: Life (Basel). 2022 Oct 30;12(11):1742. doi: 10.3390/life12111742 (PMC9698943; doi:10.3390/life12111742)
Supplement: Supplementary file 1 [file life-12-01742-s001.zip › life-1940367-supplementary.pdf]

Supplementary Material

Title: Multi-omics Analysis Reveals Clinical Value and Possible Mechanisms of ATAD1 down-regulation in Human Prostate Adenocarcinoma

Authors: Chun-Chi Chen, Pei-Yi Chu, Hung-Yu Lin

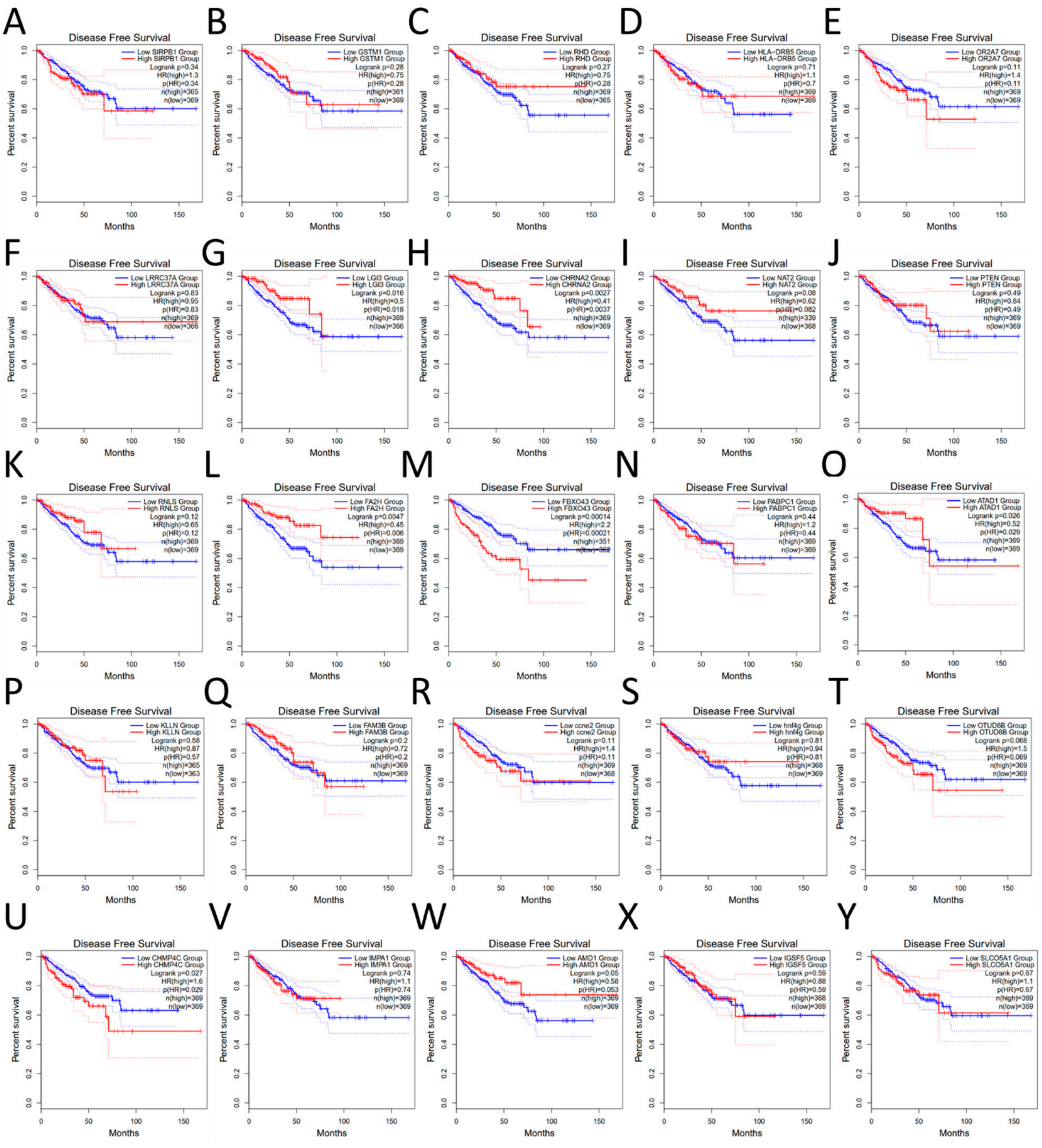

Figure S1. Kaplan–Meier survival analysis representing the probability of disease-free survival (DFS) based on high/low gene expression of PRAD patients in TCGA datasets. The cutoff vale is set at 25%–75% (high–low). Genes that exhibit CNV alterations comprise SIRBP1 (A), GSTM1 (B), RHD (C), HLA-DRB5 (D), OR2A7 (E), LRRC37A (F), LGI3 (G), CHRNA2 (H), NAT2 (I), PTEN

(J), RNLS (K), FA2H (L), FBXO43 (M), PABPC1 (N), ATAD1 (O), KLLN (P), FAM3B (Q), ccne2 (R), hnf4g (S), OTUD6B (T), CHMP4C (U), IMPA1 (V), AMD1 (W), IGSF5 (X), and SLCO5A1 (Y).

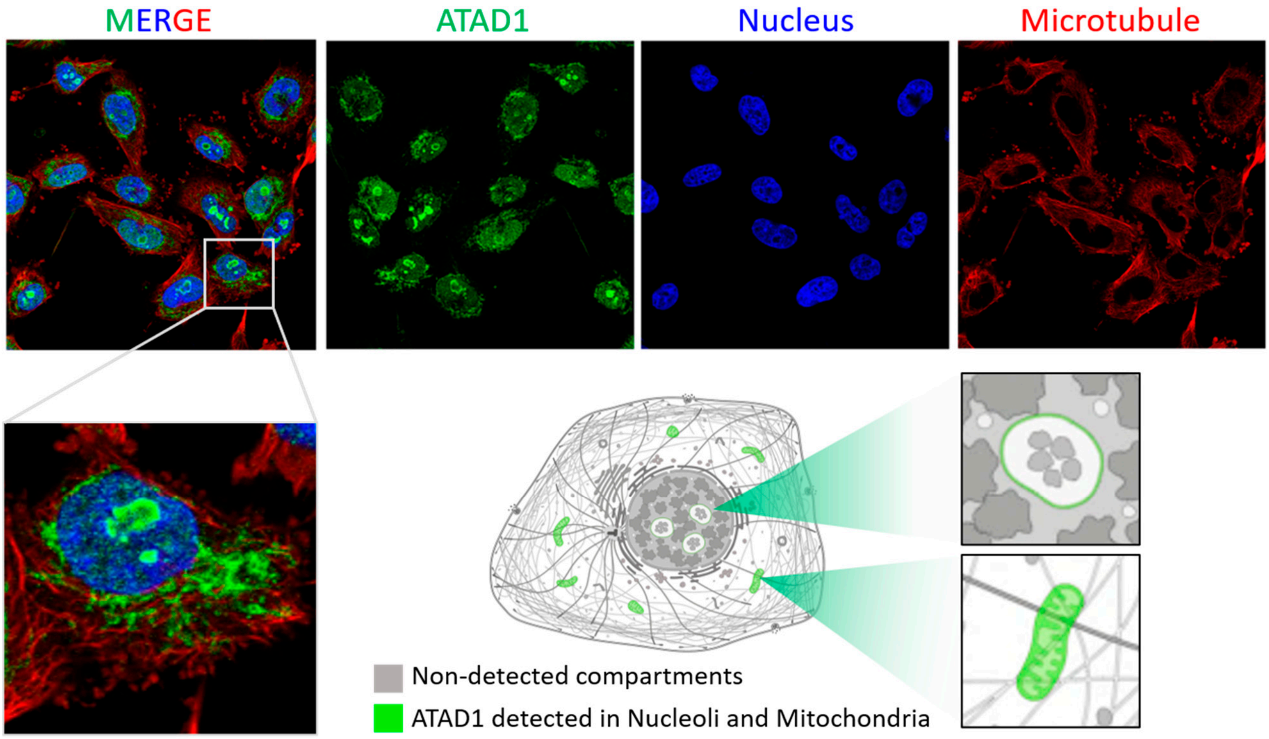

Figure S2. Representative immunofluorescence image showing intracellular localization of ATAD1 in U-251MG cells. ATAD1 (green) is probed by antibody HPA037569. Nucleus (blue) and microtubule (red) are counterstained. Bottom schematic depicts the ATAD1 localization detected in nucleoli and mitochondria.
